# Construction of A Synthetic Gene Encoding the Multi-Epitope of *Toxoplasma gondii* and Demonstration of the Relevant Recombinant Protein Production: A Vaccine Candidate

**DOI:** 10.31661/gmj.v9i0.1708

**Published:** 2020-07-20

**Authors:** Maryam Karimi, Seyyed Javad Seyyed Tabaei, Mohammad Mehdi Ranjbar, Fardin Fathi, Ali Jalili, Ghasem zamini, Amirreza Javadi Mamaghani, Javad Nazari, Daem Roshani, Nooshin Bagherani, Mohammad Bagher Khademerfan

**Affiliations:** ^1^Student Research Committee, Kurdistan University of Medical Sciences, Sanandaj, Iran; ^2^Cellular and Molecular Research Center, Kurdistan University of Medical Sciences, Sanandaj, Iran; ^3^Department of Parasitology and Mycology, School of Medicine, Shahid Beheshti University of Medical Sciences, Tehran, Iran; ^4^Razi Vaccine and Serum Research Institute, Agricultural Research, Education and Extension Organization, Karaj, Iran; ^5^Cellular and Molecular Research Center, Research Institute for Health Development, Kurdistan University of Medical Science, Sanandaj, Iran; ^6^Cancer and Immunology Research Center, Kurdistan University of Medical Sciences, Sanandaj, Iran; ^7^Department of Parasitology and Mycology, Faculty of Medicine, Kurdistan University of Medical Science; ^8^Medical Department, Arak University of Medical Science, Arak, Iran; ^9^Social Determinants of Health Research Center, Research Institute for Health Development, Kurdistan University of Medical Sciences, Sanandaj, Iran; ^10^Department of Molecular Medicine, School of Advanced Medical Sciences, Tehran University of Medical Sciences, Tehran, Iran

**Keywords:** Toxoplasma gondii, DNA Vaccine, Multi-Epitope, Bioinformatics

## Abstract

**Background:**

*Toxoplasma gondii* is a widely-distributed parasite all over the world whose attributed severe afflicting complications in human necessitate the development of serodiagnostic tests and vaccines for it. Immunological responses to monovalent vaccines and the application of diagnostic reagents including single antigens are not optimally effective. Bioinformatics approaches were used to introduce these epitopes, predict their immunogenicity and preliminarily evaluate their potential as an effective DNA vaccine and for serodiagnostic goals.

**Materials and Methods:**

A 3D structure of proteins was predicted by I-TASSER server, and linear and conformational B cell and T cell epitopes were predicted using the online servers. Then, the predicted epitopes were constructed and called Toxoeb, and their expression in the prokaryotic and eukaryotic cells was demonstrated using SDS-PAGE. In the next step, Western blotting with pooled sera of mice infected with *T. gondii* was done.

**Results:**

The current *in silico* analysis revealed that the B cell epitopes with high immunogenicity for GRA4 protein were located in the residues 34-71, and 230-266, for GRA14 in 308-387, for SAG1 in 182-195, 261-278, and for GRA7 in residues 101-120, 160-176. The T cell epitopes were selected in overlapping regions with the B cell epitopes. The immunogenic region for GRA4 are in the residues 245-253, 50-58, and 40-54, for GRA14 in 307-315, 351-359, and 308- 322, for SAG1 261-269, and 259-267, and for GRA7 in the residues 103-112, and 167-175. The results of the western blotting showed that the expressed protein had immunogenicity.

**Conclusion:**

Our constructed multi-epitope of *T. gondii* could be considered as a candidate for diagnostic and vaccination purposes.

## Introduction

*T oxoplasma gondii* is a worldwide obligate intracellular parasite that causes zoonotic diseases [1]. It is one of the most well-known protozoa due to its importance in medicine and veterinary and its propriety as a model for cellular biology investigations and molecular studies on unicellular organisms [2]. Almost one-third of people in the world are infected with this protozoa [3]. Its primary infection or recrudescence in immunocompromised individuals such as those with cancer, those undergoing chemotherapy or organ transplantation, and those with untreated HIV infection/AIDS, may lead to ocular damage and severe neurological complications which can be fatal [4,5]. The primary infections during pregnancy can result in severe complications in fetuses and infants [5]. The infection is mainly transmitted by water or food contaminated with the oocyst of *T. gondii* acquired from cat feces or by eating not cooked meat including cysts [6]. *T*. * gondii *can cause abortion and neonatal loss in livestock, mainly sheep and goat, that has economic importance [7]. Conventional medicines currently being used for the treatment of toxoplasmosis essentially have therapeutic effects on the tachyzoites, but they cannot eradicate the encysted parasites within infected hosts. Thus, the development of a suitable and effective vaccine with the ability to eradicate all forms of the parasite is of great priority that can immediately inhibit and restrict the infection in both humans and domestic animals [8]. One of the novel methods in vaccine development is the construction of synthetic multi-epitopes using the Tcell and B cells epitopes that can cause diverse T cell responses and induction of neutralizing antibodies [9,10]. Most B cell epitopes (90%) are discontinuous and more complex and difficult to be detected in comparison with linear epitopes [11,12]. Within the T cell population, the CD8^+^ T cells are the most effective cells against *T*. *gondii*, while the CD4+^+^T cells have a synergistic role in this field [13]. Studies have revealed that the immunological effects of monovalent vaccines including single antigens are not significant and perfectly effective[14]. In the past, a variety of *T. gondii* antigens have been introduced as appropriate candidates for vaccine development [15]. In different studies, it has been shown that all of the virulence factors are immunostimulatory molecules which can be considered as potential candidates for the toxoplasmosis vaccine [16,17]. On the tachyzoites, the major surface antigen1 (SAG1) has a critical role in binding and invading the host cells [15]. One of the secretory dense granule proteins, GRA4, which is present in the bradyzoites and tachyzoites and secreted by the intracellular tachyzoites, plays a key role in the survival of the parasite inside the cells [16]. GRA7 is another secretory protein that induces a strong humoral and cellular response during acute and chronic phases of infection [18,19]. GRA14 is secreted into the vacuole and traffics to both parasitophorous vacuole membrane and intravacuolar network. This protein has a specific topology with a long structure in the way to be expected that strongly can stimulate the immune system [20]. In the current study, we have exploited an immunoinformatic approach to recognize potential epitopes which could form the basis for the vaccine development against the *T*. *gondii*. According to this approach, in the current study, the T cell and B cell epitopes of SAG1, GRA4, GRA7, and GRA14 were assigned and considered together for designing a vaccine. Our objective was to introduce a multi-epitope gene with high immunogenicity as an effective candidate for vaccination against the *T. gondii*.

## Materials and Methods

###  Sequence Retrieval of Targets

Nucleotide sequences for the proteins GRA4 (Gene Bank: Q27002.1), GRA7 (Gene Bank: AFO54982.1), GRA14 (Gene Bank: ACH88354.1), and SAG1 (Gene Bank: AFO54882.1) were retrieved from the National Center for Biotechnology Information (NCBI), Nucleotide Database. Basically, the target sequences were selected from the well-known RH strain of *T*. *gondii*, which is an extremely virulent strain. Complete amino acid sequences of these four proteins were obtained from the UniProt KB database using the proteins’ ID (http://www.uniprot.org/uniprotKB/).

###  Analyzing Proteins’ Primary Structure and Physiochemical Properties 

For the analysis of primary proteins structure, amino acid sequences of the proteins were submitted to the Expasy tools. Several physicochemical parameters of proteins including the number of amino acids, instability index, molecular weight, theoretical isoelectric point (IP), grand average of hydropathicity (GRAVY), and aliphatic index were predicted using the Expasy protparam online server (http://web.expasy.org/protparam) [21].

###  Prediction of Proteins’ Secondary Structures and Transmembrane Topology

The secondary structure of the proteins was predicted using the Self-Optimized Method with Alignment (SOPMA) server (http://npsa-prabi.ibep.fr/cgibin/npsa-automat) [22]. The regions containing outside, inside and transmembrane portions of the proteins were predicted by TMHMM server 2.0 (http://www.cbs.dtu.dk/services/TMHMM) [23]. The signal peptide region of proteins was predicted by the SignalP-5.0 server (http://www.cbs.dtu.dk/services/SignalP/) [24].

###  Prediction of Proteins’ Tertiary Structures

Tertiary structures of the GRA7, GRA14, and GRA4 proteins were predicted by using I-TASSER tool [25,26]. This tool works based on multiple threading alignments and iterative model fragment assembly simulations. I-TASSER is the best tool for predicting the protein 3D structure in researches for the assessment of modeling servers. In this server, the C-score is explained as the confidence score for evaluating the quality of predicted models and higher values of the C-score illustrate the model with high confidence and vice-versa [27]. The tertiary structure of SAG1 was downloaded from PDB protein data bank (https://www.rcsb.org/).

###  Model Validation 

Models were validated by Ramachandran plots (PROCHECK; https://servicesn.mbi.ucla.edu/PROCHEC) [28].

###  Prediction of Conformational and Linear B Cell Epitopes 

For prediction of linear B cell epitopes, ABCpred (http://crdd.osdd.net/raghava/abcpred/) [29], LBtope (http://crdd.osdd.net/raghava/lbtope/protein.php) [30], SVMtrip (http://sysbio.unl.edu/SVMTriP/) [31] and IEDB(http://tools.immuneepitope.org/bcell/) web servers were used. Thus, the predictions of epitopes were mostly on the basis of Chou and Fasman beta-turn [32], Karplus and Schulz flexibility [33], Emini surface accessibility [34], Parker hydrophilicity [35], and Kolaskar and Tongaonkar antigenicity [36]. We used Kolaskar and Tongaonkar method which makes 75% accuracy in predicting linear B cell epitopes [36]. For prediction of discontinuous B cell epitopes, Discotope 2.0 (http://www.cbs.dtu.dk/services/DiscoTope/) [37] and, CBTOPE (http://crdd.osdd.net/raghava/cbtope) [38] were administered. Discotope calculation and combination connect numbers (equivalent for surface accessibility) with the tendency scores of residues in spatial proximity [37]. The CBTOPE server used the Support Vector Machine (SVM) based on a method by applying the amino acid composition produced from the query sequences with overall accuracy [38]. The result of Discotope is more valid than other conformational epitope predictor server [37]. Experimental epitopes available on the IEDB server can also be used. For epitope prediction of GRA7 and SAG1 proteins, available experimental epitopes in IEDB server were used.

###  Prediction of T Cell Epitopes 

Based on using Balb/c mouse as an animal model, the online servers IEDB (http://tools.iedb.org/main/tcell/) and SYFPEITHI (http://www.syfpeithi.de/bin/MHCServer.dll/EpitopePrediction.htm) were used for predicting alleles in the murine model [39]. The H2-Kd, H2-Ld, and H2-Dd alleles as mouse MHC-I molecules and H2-IAd and H2-IEd alleles as mouse MHC-II molecules were chosen. The T cell epitopes which showed the highest scores in relation to the mouse MHC-I and MHC-II were selected. The epitopes were also chosen based upon areas in overlapping with the B cell epitopes.

###  Construction of Epitopes

The predicted and experimental antigen epitopes were connected by linkers. The most commonly-used flexible linkers have sequences primarily consisting of stretches of glycine and serine residues [40]. Two oligonucleotide sequences with action sites for *BamHI* and *XhoI* restriction enzymes were placed at the start and endpoints of the sequence. Additionally, the sequence included a small kozak sequence, ATG start codon, and a TAG stop codon. Then, the sequence was translated into the nucleotide sequence and codon optimization was performed for eukaryotic cells (mice) by the JCAT online software (http://www.jcat.de/) (Technical University of Braunschweig, Germany) [41]. Codon optimization is a method for getting the higher expression of the foreign gene introduced by a vector in a host. The different codons of glycine and serine were used in the linkers to achieve the least repetitive codons.

###  Prediction of Designated Construct’s Structure

 Physiochemical parameters of construction including the number of amino acids, molecular weight, theoretical IP, instability index, aliphatic index and grand average of hydropathicity (GRAVY) were analyzed using the Expasy protparam online server. The secondary structure of the construct was assessed by (SOPMA) server and the tertiary structure was predicted by the online prediction I-TASSER server and was evaluated by using RAMPAGE.

###  Cloning and Protein Expression of Construct in the Prokaryotic Expression Vector 

The pUC57 plasmid harboring the inserted coding named Toxoeb was constructed and produced by a commercial supplier according to our designation (Bio Basic, Canada). In the next step, using the enzymatic digestion method, the construct was removed from the transfer vector pUC57 and subcloned in the expression plasmid pET-32a (+) (Novagen, Germany). The recombinant plasmid pET32-Toxoeb was transformed into (E. *coli*) BL21 (DE3) cells (Novagen, Darmstadt, Germany) and grown in LB broth medium (Merck, Germany) with 50µg/ml Ampicillin to an optical density at 600nm of 0.6. Expression of the recombinant protein was induced by the addition of 1mM isopropyl β-D-1 thiogalactopyranoside (IPTG) at 37°C, followed by harvesting the ( *E. coli*) BL21 (DE3) cells after 8 hours. After suspending the bacteria in the lysis buffer (Tris-HCI 50 mM, Glycerol 10%, and TritonX-100 0.1%; PH: 8), the cells were disrupted by sonication (Hielscher, UP200H, Germany) for 40 cycle 0.5 S at amplitude 70% and clarified by centrifugation for 20min at 12000×g at 4°C. The supernatant was analyzed by sodium dodecyl sulfate-polyacrylamide gel electrophoresis (SDS-PAGE; BioRad, USA).

###  SDS-PAGE and Western Blot Test

The expression of the protein was confirmed by 12% SDS-PAGE. In this process, the gel was stained with Coomassie Brilliant Blue and analyzed. The recombinant histidine-tagged pET32-Toxoeb protein was purified by Ni-NTA affinity chromatography (Qiagen, Germantown, MD, USA) according to the manufacturer’s protocols. To evaluate immunoreactivity, the purified recombinant protein was analyzed by western blot. Briefly, the protein was separated by 12% SDS-PAGE, then the gel containing protein was electro-transferred on nitrocellulose membrane. The UV Cross Linker (UV Tec, EEC) was used to stabilize the protein on the membrane. The membrane was blocked 16 h at 4°C with 3% bovine serum albumin (BSA; Merck, Germany). After washing with Tris-buffered saline and Tween 20 (TBST) three times, the membrane was incubated with pooled sera from mice infected with *T. gondii* diluted 1:50 in 1% BSA for 2 h in 37°C. Then, the membrane was re-washed three times with TBST, and was incubated with 1:5000 dilution of horseradish peroxidase (HRP) conjugated with goat anti-mouse IgG antibody at 37°C for 2 h. Thereafter, the membrane was washed and incubated with a solution containing DAB (3, 3′-diaminobenzidine; Sigma Aldrich, USA), H2O2, Tris and distilled water at RT for 15 min. At the end, the reaction was stopped using distilled water.

###  Cloning and Protein Expression of Construct in the Eukaryotic Expression Vector 

Chinese Hamster Ovary (CHO) cells and plasmid pCDNA3.1(+) were used to confirm the protein expression in the eukaryotic cells. For this reason, the CHO cells were cultivated in RPMI1640 (BioSera, France) with penicillin/streptomycin (Atocel, Hungary) and 10% fetal bovine serum (FBS; Gibco, USA). The cells were kept in a humidified atmosphere at 37°C with 5% CO2. These cells were plated in counts of 1-2×105^5^in 6 well plates. The cells were transfected using calcium phosphate [42]. After further incubation for 48 hours, the cells were harvested and suspended in lysis buffer (sodium dodecyl sulfate 1%). Then, the cells were disrupted by sonication for 20 cycle 0.5 S at amplitude 70% and clarified by centrifugation for 20 min at 12000×g at 4°C. The expression of the protein was confirmed by 12% SDS-PAGE. The gel was stained with Coomassie Brilliant Blue and analyzed.

## Results

###  Analysis of Physicochemical Properties and Transmembrane Topology

 An overview of the most important data collected from Expasy server is given in Table-1. IP is a PH point in which the net charge of the protein is zero. Its importance is in the evaluation of a proteins’ solubility and mobility in an electric field. The IP of the three proteins GRA4, GRA14, and SAG1 was greater than 7, while the IP of GRA7 was less than 7. The instability index shows an estimation of the stability of a given protein in vitro. Based on the instability index, the proteins GRA4, GRA7, and GRA14 were unstable while the SAG1 was a stable protein. The aliphatic index of the three proteins GRA7, GRA14, and SAG1 was relatively high that revealed that they had been relatively stable at different temperatures. The GRAVY index represents the degree of proteins’ hydropathicity and increasing positive scores show greater hydrophobicity. The results showed that the three proteins GRA4, GRA7, and GRA14 had high hydrophilicity, while the SAG1 compared with the three others had relatively low hydrophilicity. The aliphatic index of a given protein is defined as the relative volume which is occupied by aliphatic side chains including alanine, valine, isoleucine, and leucine. It may be considered as a positive factor for gaining thermo-stability in globular proteins. According to the aliphatic index, the proteins SAG1, GRA7, and GRA14 were stable over a wide temperature range. Assessment of the transmembrane topology revealed that the greater part of all of these four proteins were placed on the outer of the membrane.

###  Prediction of Proteins’ Structures

**Figure 1:**
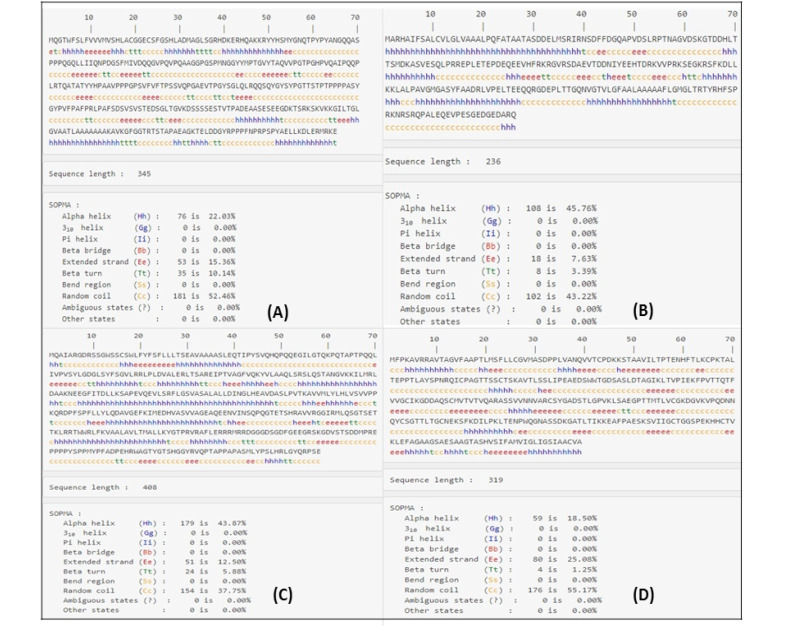
Secondary structures of proteins SAG1, GRA4, GRA7, GRA14 Toxoplasma. gondii were predicted using SOPMA server. A: GRA4, B: GRA7, C: GRA14, D: SAG1.

**Figure 2:**
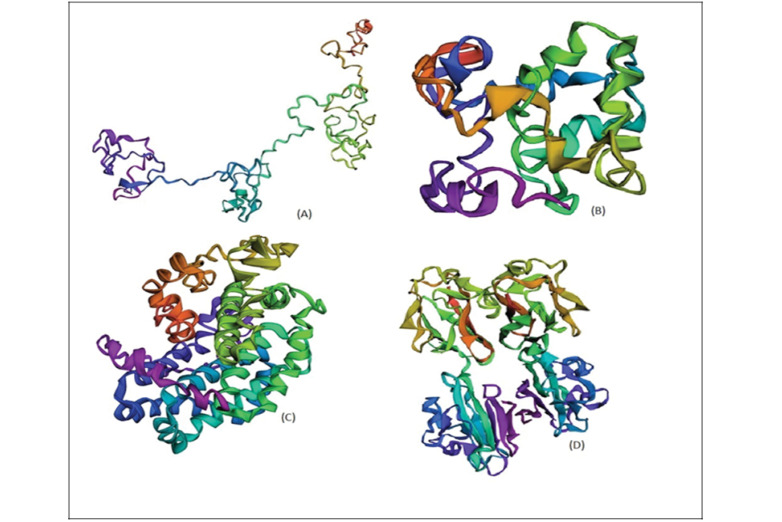
Result of Prediction of 3D Structure of Proteins SAG1, GRA4, GRA7, GRA14 Toxoplasma. gondii. Using I-TASSER and PROCHECK. A: GRA4; B: GRA7; C: GRA14; D: SAG1.

 The prediction results of the secondary structure clarified the alpha helices and beta-turn parts of the proteins. These structures were predicted using the SOPMA server (Figure-1). Tertiary structure models were generated using I-TASSER and PROCHECK server (Figure-2). C-score for the GRA4, GRA7, and GRA14 were 2.36, -3.38, and -1.92, respectively. Additionally, estimated accuracy of model 1s was 0.44±0.14 (TM-score) and 11.8±4.5A° (RMSD) for the GRA4, 0.34±0.11 (TM-score) and 12.7±4.3A°(RMSD) for the GRA7, and 0.48±0.15(TM-score) and 11.2±4.6A°(RMSD) for the GRA14. Hence, the results showed the appropriateness of these proteins for modeling.

###  Model Validation

 Evaluation of the quality of the model by PROCHECK is shown in Table-2 All of the four modeled structures were valid.

###  Prediction of Conformational and Linear B Cell Epitopes

**Figure 3:**
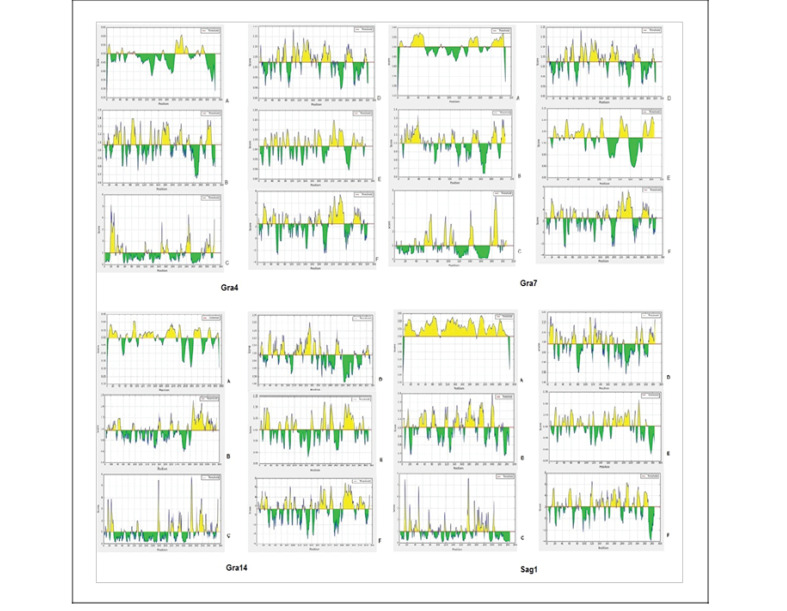
B cell Epitope Prediction for Proteins, GAR4, GRA7, GRA14, and SAG1 Toxoplasma. gondii using IEDB software. A: BepiPred linear epitope; B: Chou and Fasman β-turn; C: Emini surface accessibility; D: Kolaskar and Tongaonkar antigenicity; E: Karplus and Schulz flexibility; F: Parker hydrophilicity.

**Figure 4:**
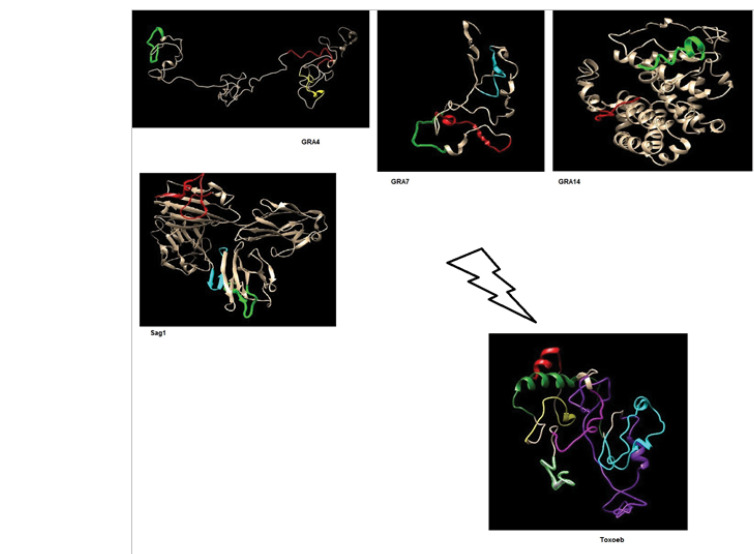
Predicted B cell Epitopes on the 3D Structures of Proteins GAR4, GRA7, GRA14, and SAG1 Toxoplasma. gondii and 3D structure of Toxoeb Construct.

 The predicted linear and discontinuous B cell epitopes are showed in Tables-3 and Figure-3. In Figure-4, the predicted B cell epitopes on the 3D structures of the proteins and Toxoeb construct are schematically shown.

###  Prediction of T Cell Epitopes 

 The predicted T cells’ epitopes are shown in Table-4. The T cell epitopes are inside the regions where the B cell epitopes are selected to simultaneously stimulate cellular and humoral immunity.

####  Construction of Epitopes

 Based on our findings, we designed an 825bp sequence DNA which we named Toxoeb. The presence of the recombinant plasmid was confirmed through digestion by the restriction enzyme, PCR and sequencing.

###  Prediction of Construct’s Structure

**Figure 5:**
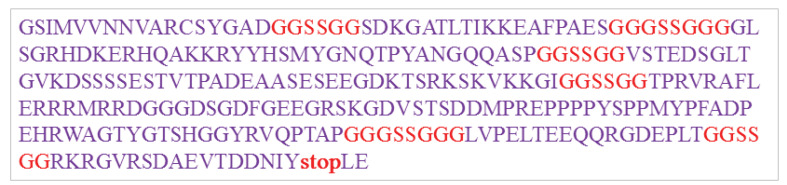
Amino Acid Sequence of Toxoeb Construct from Proteins GAR4, GRA7, GRA14, and SAG1 Toxoplasma. gondii.

 The number of amino acids of the Toxoeb construct was 275 (Figure-5). The estimated molecular weight of this construct was 30 kDa and its IP was 6.03. Its computed value was lower than 7, demonstrating the acidic nature of the protein. The aliphatic index for the construct was 34.59 indicating its instability in a wide range of temperatures. The instability index of this construct was 63.96 based upon which the construct was classified as unstable. The construct’s GRAVY index was negative (-1.08, Table-1) which revealed that Toxoeb had high hydrophilicity and tended to interact with surrounding water molecules. The result of the prediction of Toxoeb’s secondary structure showed that the ratio of α-helixes, β-turn, random coil and extended strand amount were 14.18%, 13.43%, 56.72%, and 15.67%, respectively. A high proportion of random coils in the Toxoeb construct indicated that the protein might form antigenic epitopes. The 3D structure of the construct was assessed using rampage. The result of a Ramachandran Plot showed 42.6%, 41.6%, 10.2%, and 5.6% of residues found in favored regions, additionally allowed regions, generously-allowed regions, and disallowed regions, respectively.

###  SDS-PAGE and Western Blot 

**Figure 6:**
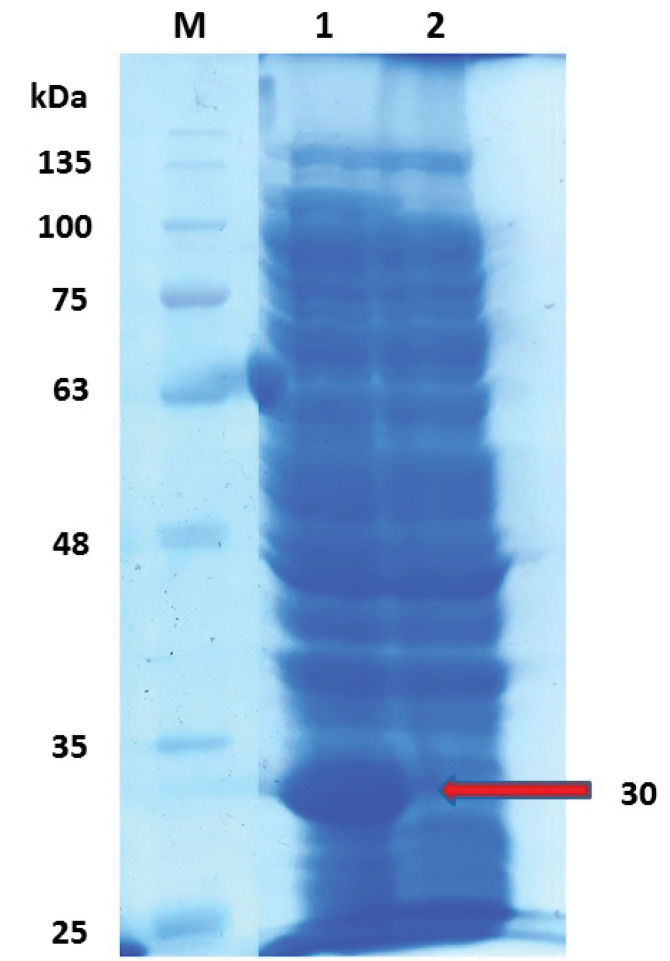
The SDS- PAGE analysis of the expression of rPet32a-Toxoeb, protein in E. Coli BL21(DE3). Lane1: expression of the rPet32a-Toxoeb protein; Lane2: BL21 containing pET-32a without the insert.

**Figure 7:**
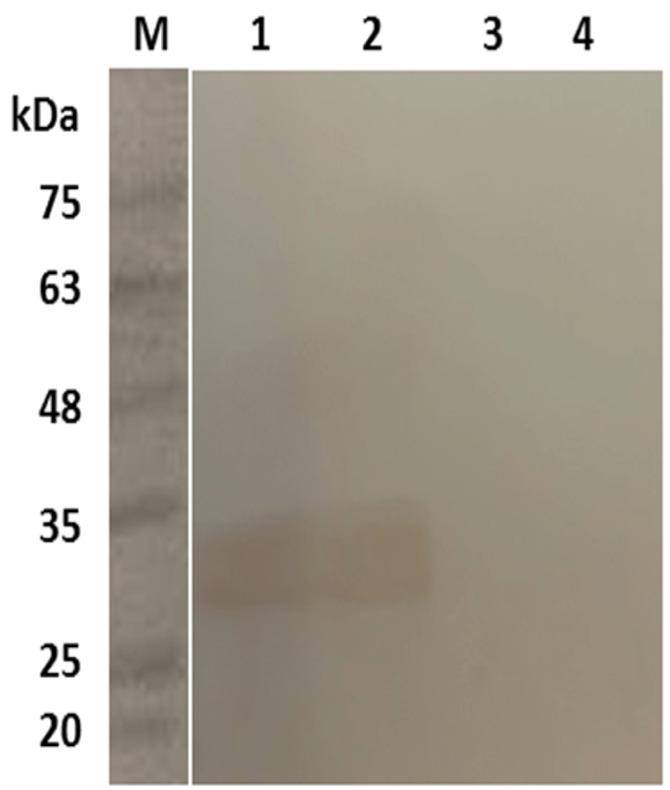
Western blotting analysis of the recombinant rPet32-Toxoeb with mice pooled sera. Lane 1 and 2: rPet32-Toxoeb; Lane 3 and 4: BL21 containing pET-32a without the insert.

**Figure 8:**
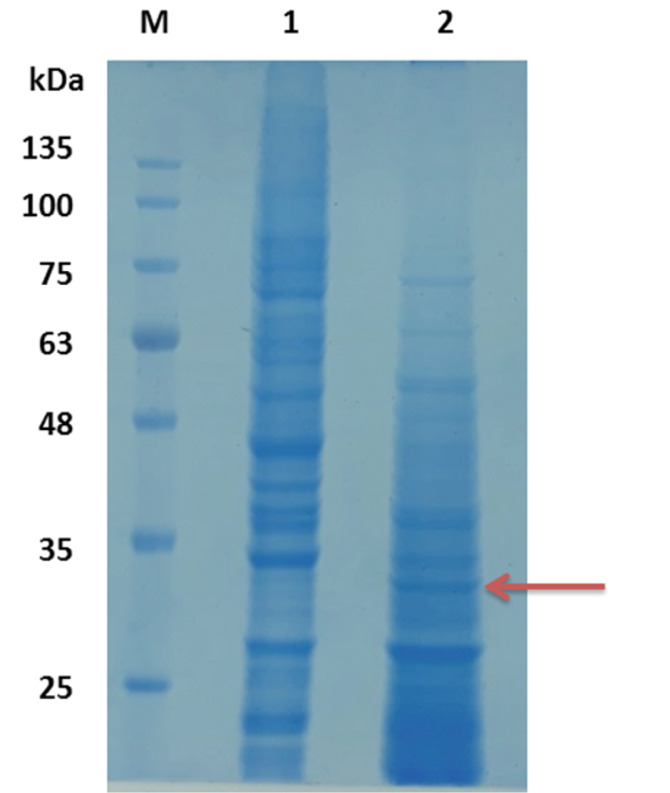
The SDS- PAGE analysis of the expression of rpCDNA3.1-Toxoeb. M: Marker; Lane 1: CHO cell containing pCDNA3.1 without insert; Lane 2: rpCDNA3.1-Toxoeb.

 Results of the SDS-PAGE for the recombinant pET32a-Toxoeb showed a single protein band with a molecular weight of 30kDa representing the protein expression (Figure-6). Western blotting with a 1:50 dilution of mice sera demonstrated that the expressed proteins had had immunogenicity, whereas the control intact plasmid pET-32a (+) show no any band (Figure-7). The SDS-PAGE for the recombinant pCDNA3.1-Toxoeb showed a band representing the protein expression in 30kDa, whereas the intact plasmid pCDNA3.1 shows no any band (Figure-8).

## Discussion

Immunological research has shown that antigens do not function through their entire molecule; their proprietary epitopes are the source of the immune response [43]. Epitopes are chemical groups that introduce the features of a given antigen [44]. The structure of protein antigens is not only made of specific epitopes used by the B, T, and cytotoxic T lymphocytes, and NK cells for triggering immunological responses, but they may also have structures not required for the immune system to induce protective responses. Therefore, studies on these epitopes have increased our knowledge of the structure and activity of antigens, the antigen-antibody interactions, and other immune system activities that play an important role in developing new diagnostic reagents and vaccines [43]. Studies have revealed the immunological responses to monovalent subunit vaccines not to be optimal, and diagnostic reagents consisting of single antigens not to be desirable [45]. The application of immunoinformatic methods for the prediction of immunogenic epitopes has achieved a good place as an essential instrument for epitope localization. Bioinformatics, as an interdisciplinary science, has been widely applied to predict protein function, structure, and epitopes [46]. These methods have decreased blind points in epitope identification, and have improved the accuracy and precision of this identification. Additionally, such methods are efficient, economically appropriate, and able to decrease the need for experimental instruments [47]. Given the subsequent considerable progress in bioinformatics, a diversity of parameters containing a scheme of hydrophilicity, flexibility, accessibility, and antigenicity have been developed [32-34,48,49], which have played a significant role in enhancing studies on linear epitopes. To improve the prediction of epitopes, it is often essential to combine a variety of algorithms and findings from the multi-level analysis [50]. Combination of computerized prediction algorithm and experimental tools have resulted in the fast development of conformational epitope analysis and localization, and based on these combinations, several effective prediction programs have been released [43]. The success of polytope vaccines’ development is related to rigid criteria for choosing epitopes and also the linkers between these epitopes [9]. Obtaining information regarding antigen epitopes would facilitate the development of epitope vaccines. Previously, the epitope prediction was performed based on only a single parameter, while currently, its accuracy has been limited secondary to progressing development in the bioinformatics field. Nowadays, by using a multi-parameter and multi-method analysis, the accuracy of epitopes’ prediction has significantly been improved. Primarily, many researchers all over the world, who are working on the production of vaccines and diagnostic reagents, design and assess them in silico by using bioinformatics tools to reduce the cost for their production, lessen blind spots, and increase the chance of success. Shan Liu *et al*. developed a multi-epitope DNA vaccine expressing the 6 antigen segments from *T. gondii*, which was used for immunizing mice through its introduction by the recombinant plasmid. The authors reported high survival rates in mice when they were challenged with the *T. gondii* RH strain [51]. In another study, Hajissa *et al*. assessed the protective efficacy of a recombinant multi-epitope antigen-expressing nine epitopes from *T*. *gondii *in mice. They concluded that the multi-epitope recombinant antigen-induced strong immunity against the T. *gondii* RH strain [52]. In the current study, we also firstly designed the vaccine candidate construct by accessible software, and then we evaluated its immunogenicity in vitro. Based on our schedule, the next step will be to study the immunogenicity of this gene in the murine model. In the current study, the B cell epitopes GRA4, GRA7, GRA14, and SAG1 were fully analyzed using online services. For the prediction of the conformational B cell epitopes, knowing the spatial structure of proteins is essential. The 3D structure of some proteins has been determined by methods such as crystallography. In the case of proteins whose 3D structure is unknown, their structures would be predicted only based on sequencing and checking the forces, but not using experimental structural information. One of the limitations of our study was the inaccessibility of the actual tertiary structures of GRA7, GRA4, and GRA14 proteins; thus, we had to predict probable tertiary structures using some bioinformatics tools. Inaccessibility to some of the prediction software currently accessible for researchers all over the world, because of our sanctions, was another limitation of our study. Prediction of the linear B cell epitopes was performed using the LBTOPE, SVMtrip, ABCpred, and IEDB; and for conformational B cell epitopes using CBTOPE and Discotope server. Based on the prediction, we concluded that for the GRA4 protein, the residues in the regions 34-71 and 230-266, for the GRA14 protein in 308-387 and 308-322, for SAG1 in 182-195 and 261-278, and for GRA7 in 101-120 and 160-176 had the most immunogenic potential. Prediction of the T cell epitopes was carried out by the IEDB and SYFPEITHI servers. The immunogenic regions for the GRA4 protein were the residues of 245-253, 50-58, and 40-54, for the GRA14 protein in the residues of 307-315, 351-359, and 308-322, for the SAG1 in the residues of 261-269, and 259-267, and for the GRA7 in the residues of 103-112 and 167-175. These regions had decent potential for designing epitopes. The T cell and B cell epitopes were selected in the overlapping regions. The selected epitopes were connected by linkers. The flexible linkers are usually used when the attached domains need a certain degree of mobility and interplay. They are commonly composed of small, non-polar ( *e.g.,* glycine) or polar ( *e.g.,* serine or threonine) amino acids. The small size of these amino acids enables inflexion and permits for movement of the joined functional domains [40]. Using the UCSF Chimera1.8.1 software (Resource for Biocomputing, Visualization, and Informatics University of California, USA), epitopes and their positions were identified on the 3D structures, and β-turn and α-helices, respectively, and their accessibilities were viewed. Their sequences were translated into the nucleotide one by the JCAT online software. Additionally, the codon optimization was performed for the eukaryotic cells (mice) by this software. Codon usage bias refers to differences in the frequency of synonymous codon occurrence in coding DNA. Although codon optimization was performed for the eukaryotic cells, the sequence was sub-cloned in the prokaryotic plasmid, and finally, its expression was demonstrated. Additionally, its expression in the eukaryotic plasmid, which had already been confirmed by SDS-PAGE, was verified in immunogenicity by the western blotting of the infected mice sera. Western blotting on the mice pooled sera showed that the expressed protein had immunogenicity and can be used for diagnostic purposes and vaccine development.

## Conclusion

 The current paper describes the primary, secondary, and tertiary structure of the proteins GRA4, GRA7, GRA14, and SAG1; and their T cell and B cell epitopes using bioinformatics approaches and online servers. An 825 bp sequence DNA was constructed and its immunogenicity was confirmed in vitro. In conclusion, this multi-epitope synthetic gene can be used for the development of DNA vaccine and diagnostic reagents.

## Acknowledgment

 This paper is a part of the Ph.D. thesis of the first author and was supported by a research grant (code: IR.MUK.REC.1396/116) from Kurdistan University of Medical Science. The authors would like to appreciate very much for kind collaboration of Prof. Bruce R. Smoller, Professor and Chair, Department of Pathology and Laboratory Medicine, Professor, Department of Dermatology University of Rochester School of Medicine and Dentistry.

## Conflict of Interest

 None declared.

**Table 1:** Parameters Computed for Proteins and constructed gene from Proteins SAG1, GRA4, GRA7, GRA14 Toxoplasma. gondii Using by Expasy Protparam Tool.

**Parameters**	**SAG1**	**GRA4**	**GRA7**	**GRA14**	**Toxoeb**
**No. of amino acid**	319	345	236	408	275
**Molecular weight (KD)**	32.6	36	25.8	44.7	30
**Theoretical pI**	7.43	7.05	5.09	8.49	6.03
**Instability index**	36.47	51.84	50.96	52.86	63.96
**Aliphatic index**	79.84	56.32	69.96	85.59	34.59
**GRAVY**	0.111	-0.467	-0.615	-0.213	-1.08

**GRAVY: **Grand average of hydropathicity

**Table 2:** Ramachandran Result of the Predicted model from Proteins SAG1, GRA4, GRA7, GRA14 Toxoplasma. gondii.

**Residue **	**GRA4**	**GRA7**	**GRA14**	**SAG1**
Residue in most favored regions	36.4%	28.7%	70%	87.6%
Residue in most allowed regions	56%	65.2%	27%	12.4%
Residue in most disallowed regions	7.5%	5.9%	2.6%	0%

**Table 3:** Predicted B Cell Epitopes and Selected Final Consensus Epitope from Proteins SAG1, GRA4, GRA7, GRA14 Toxoplasma. gondii.

**Protein ID**	**Designed B cell epitope**	**Residue**	Status
SAG1 (Gena Bank: AFO54882.1)	SDKGATLTIKKEAFPAES	261-278	Experimental
	SVVNNVARCSYGAD	182-195	Experimental
GRA7 (Gena Bank: AFO54982.1)	RKRGVRSDAEVTDDNIY	101-120	New
	LVPELTEEQQRGDEPLT	160-176	Experimental
GRA4 (Gene Bank: Q27002.1)	VSTEDSGLTGVKDSSSSESTVTPA- -DEAASESEEGDKTSRKSKVKKGI	230-266	New
	GLSGRHDKERHQAKKRY- -YHSMYGNQTPYANGQQASP	34-71	New
	GRA14 (GenBank: ACH88354.1)	TPRVRAFLERRRMRRDGGGDSGDFGEEGRSKGDVSTSDD- -MPREPPPPYSPPMYPFADPEHRWAGTYGTSHGGYRVQPTAP	308-387	New

**Table 4:** Predicted T Cell Epitopes from SAG1, GRA4, GRA7, GRA14 Toxoplasma. gondii

**Protein ID**	**Designed T cell epitope**	**Residue**
SAG1 (Gena Bank:AFO54882.1)	SDKGATLTI	261-269
	ASSDKGATL	259-267
GRA7 (Gena Bank:AFO54982.1)	RGVRSDAEV	103-112
	EQQRGDEPLT	167-175
GRA4 (Gene Bank:Q27002.1)	SSESTVTPA	245-253
	YYHSMYGNQ	50-58
	DKERHQAKKRYYHSM	40-54
GRA14 (GenBank:ACH88354.1)	GTPRVRAF	307-315
	PPPPYSPPM	351-359
	TPRVRAFLERRRMRR	308-322
